# Editorial: Avian Muscle Development and Growth Mechanisms: Association With Muscle Myopathies and Meat Quality Volume II

**DOI:** 10.3389/fphys.2021.765515

**Published:** 2021-10-14

**Authors:** Massimiliano Petracci, Sandra G. Velleman

**Affiliations:** ^1^Department of Agricultural and Food Sciences, Alma Mater Studiorum – University of Bologna, Bologna, Italy; ^2^Department of Animal Sciences, The Ohio State University, Wooster, OH, United States

**Keywords:** meat, myopathies, poultry, spaghetti meat, white striping, wooden breast

Given the significant interest in Volume I, it was decided to launch Volume II of the Research Topic “*Avian Muscle Development and Growth Mechanisms: Association With Muscle Myopathies and Meat Quality*.” The broiler industry is still facing an unsustainable occurrence of growth-related muscular abnormalities that mainly affect fast-growing genotypes selected for high growth rate and breast yield. From their onset, research interest in these issues continues as proven by the temporal trend of published papers during the past decade (Figure 1). Even if meat affected by white striping, wooden breast, and spaghetti meat abnormalities is not harmful for human nutrition, these conditions impair quality traits of both raw and processed meat products causing severe economic losses in the poultry industry worldwide (Petracci et al., 2019; Velleman, 2019). Since the Research Topic of “*Avian Muscle Development and Growth Mechanisms: Association With Muscle Myopathies and Meat Quality*” is quite diverse, contributions in this second volume reflect the broad scope of areas of investigation related to muscle growth and development with 11 original research papers and one mini-review from prominent scientists in the sector. We hope that this collection will instigate novel questions in the minds of our readers and will be helpful in facilitating the development of the field.

**Figure 1:**
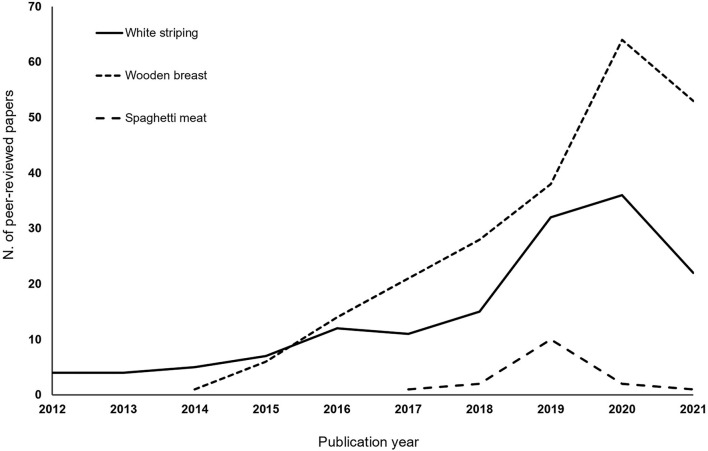
Number of publications produced per year containing concerning white striping, wooden breast, and spaghetti meat myopathies affecting broilers' pectoralis major muscles. Data obtained from Scopus on August 27, 2021.

The majority of papers included in this Research Topic further investigated possible mechanisms directly and indirectly involved in the development of growth-related broiler breast abnormalities. Recently, an extensive review paper confirmed that myopathic disorders affecting pectoral muscles of fast-growing broilers have a complex etiology, and several biological pathways, as well as response mechanisms, are involved in their progression (Soglia et al., 2021). Aside from distinctive phenotypes of white striping, wooden breast, and spaghetti meat, these conditions share common histological features. Hence, it seems that common causative mechanisms are responsible for the physiological and structural perturbations commonly detected in muscles showing these conditions and might underpin their appearance (Soglia et al., 2021). The narrative mini-review by Baldi et al. provides a critical evaluation of the state of research on the spaghetti meat condition which is one of the more recent muscular abnormalities affecting the breast muscles of fast-growing broilers. Due to its recentness, the causative mechanisms are still partially unknown and less investigated than wooden breast and white striping abnormalities (Figure 1). This mini-review highlights knowledge gaps and unexplored areas including a critical view on current uncertainties and future directions. To deepen the knowledge on the genetic mechanisms involved in white striping, Marciano et al. evaluated by quantitative PCR a selected group of 15 functional candidate genes in breast muscles of normal and white striping-affected broilers. Among them, six genes (CA2, CSRP3, PLIN1, CALM2, DNASE1L3, and MYLK2) associated with myogenic and calcium signaling were differentially expressed. These findings confirmed that dysregulation of the muscle and calcium signaling pathways plays a role in the development at least of white striping in broilers. Malila et al. evaluated transcriptional profiles associated with the wooden breast condition in broilers slaughtered at two commercial slaughter ages. The analysis of up and downregulated transcripts in wooden breast samples in comparison with normal counterparts showed that an abnormal remodeling of an extracellular matrix focal adhesion mediated the signaling pathway. In addition, remarkable perturbations in the glucose and lipid metabolic and signaling pathways are of utmost importance in broilers at 6 weeks of age, while failure of muscle regeneration was more evident in birds slaughtered at 7 weeks of age. On the other hand, Xing et al. investigated the potential physiological alterations and the possible related mechanisms in the chicken liver in order to explain its possible involvement in wooden breast occurrence. The primary findings revealed that wooden breast-affected birds exhibited clear clinical signs of liver injury associated with reduced antioxidant capacity, mitochondrial dysfunction, hepatocyte apoptosis, and a higher inflammatory response, which possibly contribute to the aggravation of liver injury in WB myopathic birds.

The paper of Ran et al. focused on DNA methylation which is a crucial epigenetic mechanism associated with skeletal muscle development during the embryonic period and exerts a key role during the early post-hatch period in broiler chickens. It was revealed that the many differentially methylated genes were strongly involved in several aspects related to embryonic muscle development. The CFL2 gene has been specifically screened for effects on satellite cells *in vitro* and demonstrated a remarkable function as a negative regulator of muscle satellite cell proliferation by leading to an induction of apoptosis. On the same topic, Orlowski et al. investigated the implications of selection for high breast-yield during the period of hyperplastic growth in order to ascertain if the number of muscle fibers is changed. For this study, two chicken lines after five generations of divergent selection for breast yield were used and sampled at different stages (late embryonic development, post-hatching, and commercial slaughter age). The high breast-yield line showed a greater rate of muscle fiber formation, while at market age, greater breast development was merely due to a larger fiber diameter as no differences were detected between the lines for fiber number. The paper of Xu et al. explored how temperature and growth selection can influence intracellular lipid accumulation and adipogenic gene expression in turkey pectoralis major muscle satellite cells. It was found that heat stress impairs the adipogenic potential of turkeys even at 7 days of age and increased the intracellular lipid content in pectoralis major muscle satellite cells during proliferation, while cold temperature exposure showed a reverse effect.

The research paper of Zhang et al. is conversely devoted to evaluating the progression of protein expression profiles in breast muscle of a native Chinese chicken breed in the final stage of growth prior to slaughter (90, 120, and 150 days) by using a proteomic approach (namely tandem mass tag). Overall, the main findings revealed that differentially expressed proteins among different ages were especially associated with carbohydrate metabolism, adrenergic signaling in cardiac myocytes, focal adhesion, oocyte meiosis, and phagosomes. These last couple of studies can help to unravel the molecular mechanisms of economically important traits in chickens.

The papers of Liu et al. and Métayer-Coustard et al. focused on the biological control glycogen content in muscle which can affect body homeostasis, live performance, and final meat quality. Liu et al. investigated the poorly explored role of FOS-like 2 AP-1 transcription factor subunit (FOSL2) in the regulation of muscle glycogen content in poultry. It was revealed that the FOSL2 gene could regulate the amount of glycogen stored in skeletal muscles through their influence on a downstream gene (CEBPB), and small differences were detected when effects of breed and gender were assessed. Métayer-Coustard et al. used chicken lines divergently selected on ultimate pH as a model to generate new knowledge on molecular mechanisms linked to energy metabolism and protein synthesis during the early post-hatch period. The primary findings revealed that muscle metabolic differences in chicken lines divergently selected are already well evident at hatch, and lower muscle glycogen storage potential is associated with higher protein synthesis rates, muscle growth, and proportion of white striping cases in the pectoralis major muscle. Further studies are needed to understand if changes observed at hatching could be due to differences in nutrient use or availability during embryonic development.

Two papers in our Research Topic focused on biomarkers for the early prediction of growth-related abnormalities in live birds with an emphasis on wooden breast. Currently, wooden breast–affected birds are phenotypically detected especially at the breeder level through manual palpation of the pectoralis major muscle. Kong B. et al. examined blood plasma proteins and metabolites in chickens at 4 and 8 weeks of age to identify early-age biomarkers for the wooden breast abnormality using untargeted metabolomics based on gas chromatography coupled with time-of-flight mass spectrometry. Among the 32 examined metabolites, raffinose, and 3-hydroxybutyric acid were reported to have the highest fold-changes in chickens showing the wooden breast condition, and their concentrations were also shown by targeted biochemical assays. In addition, Kong F. et al. assessed enzyme activity in serum and breast tissue and compression force of raw breast filets in broilers at 4 and 6 weeks of age. The main findings evidenced that creatine kinase activity in both serum and breast tissue was much higher in wooden breast–affected birds even if only in birds of 6 weeks of age. Furthermore, a linear relationship between creatine kinase levels and compression force was found. Overall, both studies have been able to identify candidate biomarkers for the prediction of the wooden breast condition and to assist genetic selection in fast-growing broiler breeding.

There is also the need for objective methods to detect and grade abnormal breasts after deboning in commercial chicken processing plants. Currently, wooden breast meat is usually sorted out by visual evaluation and manual hand-palpation. Siddique et al. assessed bioelectrical impedance analysis combined with advanced data analytics techniques such as supervised learning algorithms as an objective tool to identify wooden breast filets. Machine learning is already effectively used in the food industry (i.e., quality control, processing optimization) and this first study evidenced that supervised learning algorithms can also assist to accurately sort breasts filets into myopathy categories.

The last paper by Kuttappan et al. explored possible nutritional intervention strategies to reduce the incidence of wooden breast. Their selected approach was to test dietary supplementation of feed additives with potential antioxidant effectors such as ethoxyquin, combination of chelated trace minerals (Zn, Cu, and Mn), and organic selenium in three independent experiments. Overall, it was proven that the dietary use of antioxidants and chelated trace minerals was able to reduce oxidative stress in the tissue and reduce the severity of wooden breast.

## Author Contributions

MP wrote the first draft. SGV added notes and revised the manuscript. All authors contributed to the article and approved the submitted version.

## Conflict of Interest

The authors declare that the research was conducted in the absence of any commercial or financial relationships that could be construed as a potential conflict of interest.

## Publisher's Note

All claims expressed in this article are solely those of the authors and do not necessarily represent those of their affiliated organizations, or those of the publisher, the editors and the reviewers. Any product that may be evaluated in this article, or claim that may be made by its manufacturer, is not guaranteed or endorsed by the publisher.
